# A Rare Association of Patent Ductus Arteriosus (PDA) With Persistent Left Superior Vena Cava (PLSVC) and Unroofed Coronary Sinus (UCS) Terminating Into Left Atrium (LA): A Case Report of an Indian Infant

**DOI:** 10.7759/cureus.30124

**Published:** 2022-10-10

**Authors:** Isha Sahai, Benumadhab Ghosh, Gajendra Agrawal, Johann Christopher, Tarun Rao

**Affiliations:** 1 Cardiology, Jawaharlal Nehru Medical College, Datta Meghe Institute Of Medical Sciences, Wardha, IND; 2 Cardiology, Jawaharlal Nehru Medical College, Datta Meghe Institute of Medical Sciences, Wardha, IND; 3 Cardiology, CARE Hospital, Hyderabad, IND

**Keywords:** persistent left superior vena cava (plsvc), murmurs, unroofed coronary sinus (ucs), patent ductus arteriosus (pda), congenital heart defect (chd)

## Abstract

Unroofed coronary sinus syndrome (UCSS), also named coronary sinus (CS) septal defect, is a rare type of atrial septal defect (ASD) with the incidence less than 1% of the total number of ASDs. It is caused by incomplete formation of left atrial venous folds during embryonic development. There is communication between the CS and left atrium (LA) due to the presence of a left superior vena cava (LSVC) along with an incomplete or complete loss of the CS roof draining into the LA. It usually presents as mild breathlessness on exertion and the appearance of murmurs on auscultation. A case that is diagnosed as an unroofed CS (UCS) related to a continuous LSVC terminating into the CS, which further terminates into LA, along with a large patent ductus arteriosus (PDA) is a rare presentation.

## Introduction

Venous drainage that drains into the right atrium (RA) of the human heart comprises singular superior vena cava (SVC), single inferior vena cava (IVC), and a coronary sinus (CS). There are cases in which there are dual SVC [1]. The development of veins from the pulmonary and systemic origin is complicated and subjected to embryological variation. The superior systemic venous channels arise from the symmetrical cardinal veins, whereas the pulmonary venous channels come from the plexus supplying the foregut known as the splanchnic plexus [2]. In many cases, the left side of the cardinal system disappears. Thus, the cardiac veins are drained by the CS along with its remnant called the ligament of Marshall [2,3]. There are several variations in the development of venous structures. One among them is the presence of a left SVC (LSVC) [4], which mostly ends in the CS. This is an infrequent, unusual condition in which the LSVC is persistent. It pours directly in the left atrium (LA) [5,6]. The prevalence rate of persistent LSVC (PLSVC) is 0.1% to 0.3%, as described by Eldin et al. [7]. A PLSVC and a roofless CS is an unusual genetic heart disease that is quite rare. It was Raghib et al. who put forward this condition for the first time in 1965 [6]. There are four types of this roofless CS. In type I, an association of PLSVC is present and unroofed; in type II, the CS is also completely unroofed but the PLSVC is absent; in type III, partial unroofing is observed in the middle of the CS; and type IV shows a partial unroofing at the terminal portion of the CS [8,9]. The clinical diagnosis of this condition is vital to the patient's prognosis as there can be an embolism in the cerebrum or an abscess in the brain resulting from a shunt directed from right to left. These patients tend to have disproportionate pulmonary arterial hypertensive features, which are unexplained despite intracardiac shunts. However, clinical diagnosis in most cases is difficult due to the undetailed clinical features [9].

This case report describes a clinical scenario of a one-year-old patient. The patient visited the Pediatric Cardiology department of the Acharya Vinoba Bhave Rural Hospital (AVBRH), under Datta Meghe Institute of Medical Sciences, Wardha, India, presented with LSVC draining into the CS, which further drains into the LA with the appearance of a large patent ductus arteriosus (PDA). This is a rare case scenario, and its clinical consequences might be significant.

## Case presentation

A one-year-old child was brought by his father to the out-patient department (OPD) in a conscious state with no active complaints for a regular health checkup. During the routine health checkup, the pediatrician heard continuous grade 4 murmurs (loud and associated with palpable thrill) during auscultation. The clinician referred the patient to the pediatric cardiology department of AVBRH, where the patient was immediately advised to undergo a 2D Echo and CT angiography. The patient has no history of vomiting. The patient has no history of loose stools. The patient has no history of fever, cough, or cold. The biventricular functions were normal. At the time of birth, the neonate weighed around 2.6 kg. There was no history of NICU stay and did not show a delay in the development profile. All the milestones have been achieved to date. The patient has been immunized as per national immunization schedule (NIS). Anthropometric history shows a normal growth pattern of the patient as per WHO standards.

On general examination, the patient was conscious and well oriented with time, place and person. The patient seemed afebrile, pulse rate was around 162 /min, respiratory rate was recorded at 36/ min, and blood pressure was measured and was found to be normal (110/70 mmHg). The oxygen saturation was 98% (SpO2). There were no other significant findings in the general examination. A clinical examination revealed normal findings of the central nervous system (as per Glasgow coma score and reflex examinations), respiratory system, gastrointestinal system, and renal system. Cardiovascular systems showed abnormalities. On the CNS examination, the patient was conscious and well-oriented. Respiratory examination showed air entry bilaterally equal (AEBE). On cardiovascular assessment, no precordial bulge was seen, apex impulse was visible, and there were no dilated veins, scars, or sinuses. On palpation, the presence of a thrill was heard on the apex beat over the fifth intercostal space and anterior axillary line. Both heart sounds, S1 and S2, were heard on auscultation, along with a continuous murmur. The abdominal assessment showed a soft abdomen. There was an absence of any kind of tenderness or distention over the abdomen. There was no hepatomegaly associated. 

**Figure 1:**
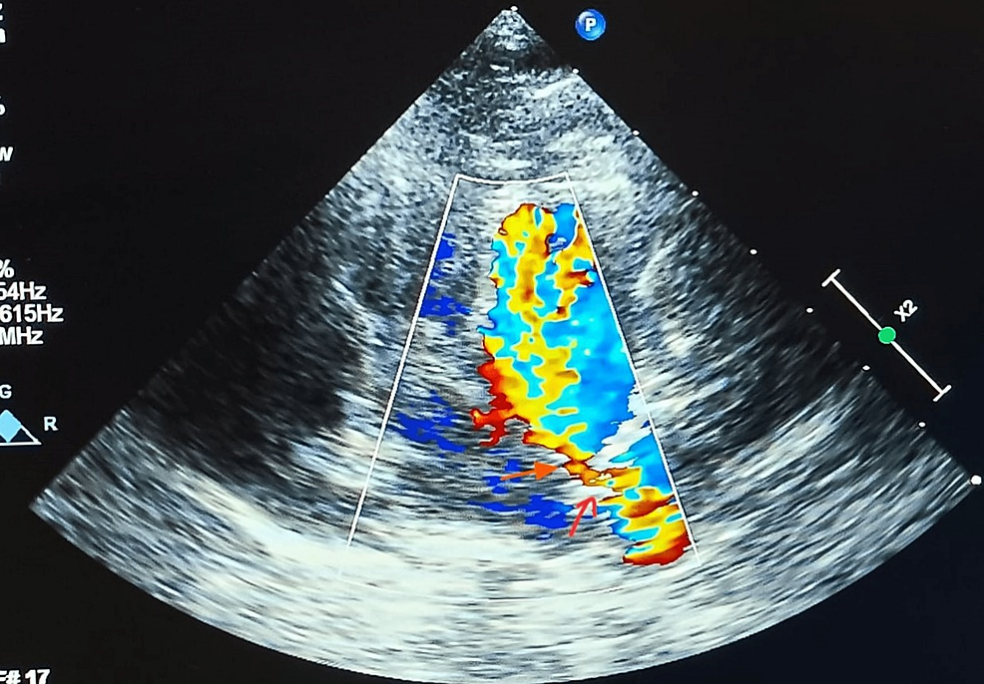
2D Echo: Parasternal Short Axis View Showing PDA PDA: Patent ductus arteriosus The image has been taken by the authors.

Lab findings show normal sodium levels (139 mEq/dl), normal potassium (4 mEq/dl), and low serum creatinine (0.3 mg/dl). Hematological findings showed normal hemoglobin levels (11.5 g%) and normocytic normochromic red blood cells. Upon ultrasonography (USG), it was observed that the liver had normal shape, size, and echo texture and had no focal lesion; the gallbladder appeared normal descendent; the pancreas was normal in size, shape, and texture; and the spleen was normal. Renal system findings revealed normal size and shape of both kidneys without any focal lesions or calculus. The urinary bladder appeared normal. Echocardiography was performed, which revealed that the patient had an enlarged ostium of the CS, which was shunting left to right. There was an unroofing of the terminal portion of the CS, where the left atrial blood entered the dilated CS, a large PDA, an enlarged LA, and an enlarged LV (Figure 1).

**Figure 2:**
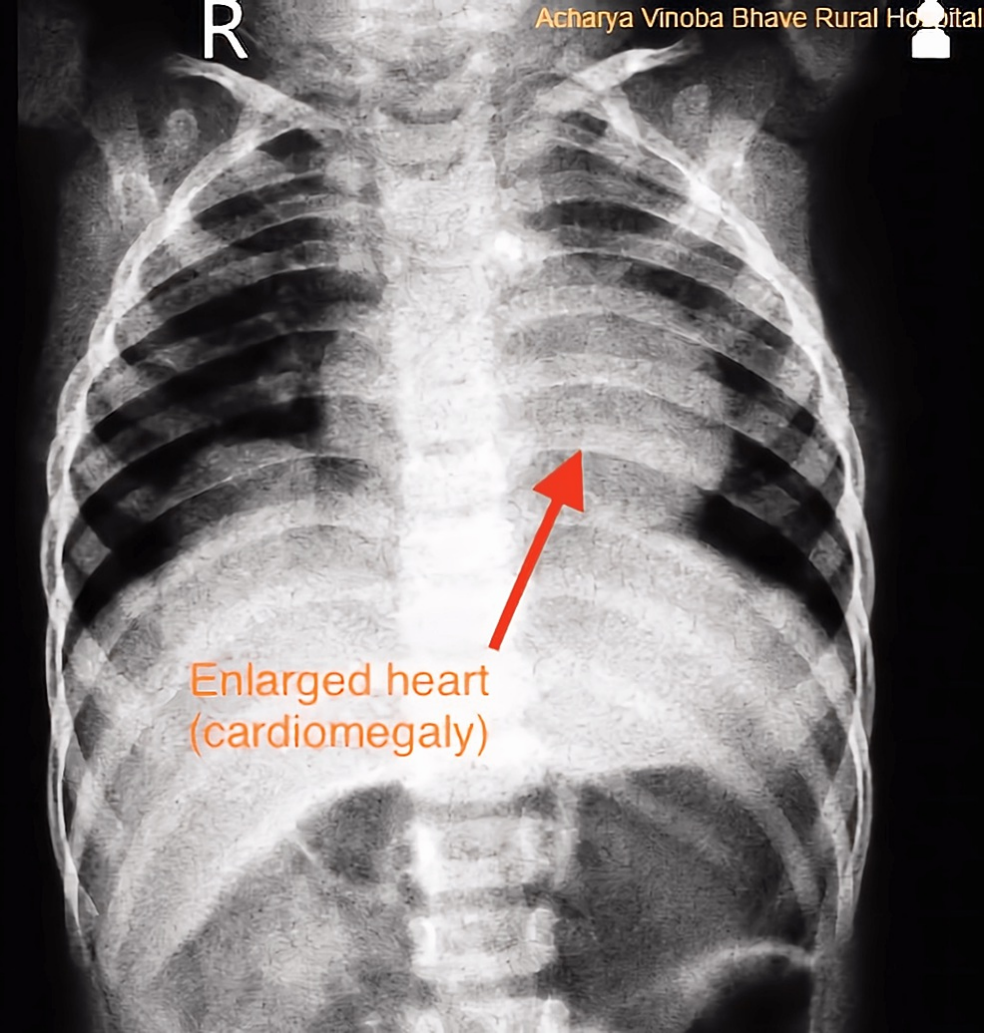
Chest X-Ray Showing Cardiomegaly The image has been taken by the authors.

A chest X-ray was performed, which revealed cardiomegaly. The left portion of the heart was enlarged, causing a reduction in the lung structure of the patient (Figure 2).

**Figure 3:**
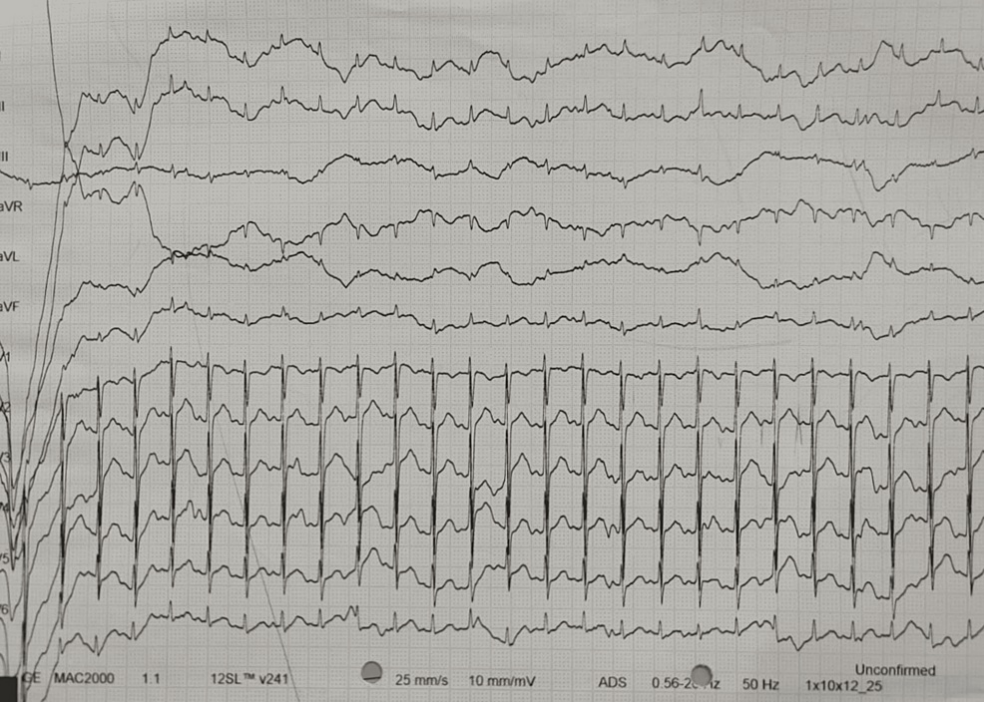
ECG Showing Normal Readings ECG: Electrocardiography The image has been taken by the authors.

Electrocardiography (ECG) shows normal findings with the PR interval is seen to be around 120 milliseconds and the QRS complex being about 120 milliseconds (Figure 3).

**Figure 4:**
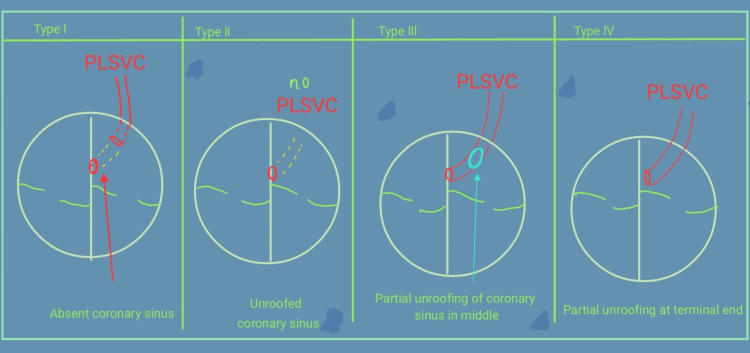
Comparing Different Types of LSVC LSVC: Left superior vena cava; PLSVC: Persistent left superior vena cava The diagram has been created solely by the authors.

**Figure 5:**
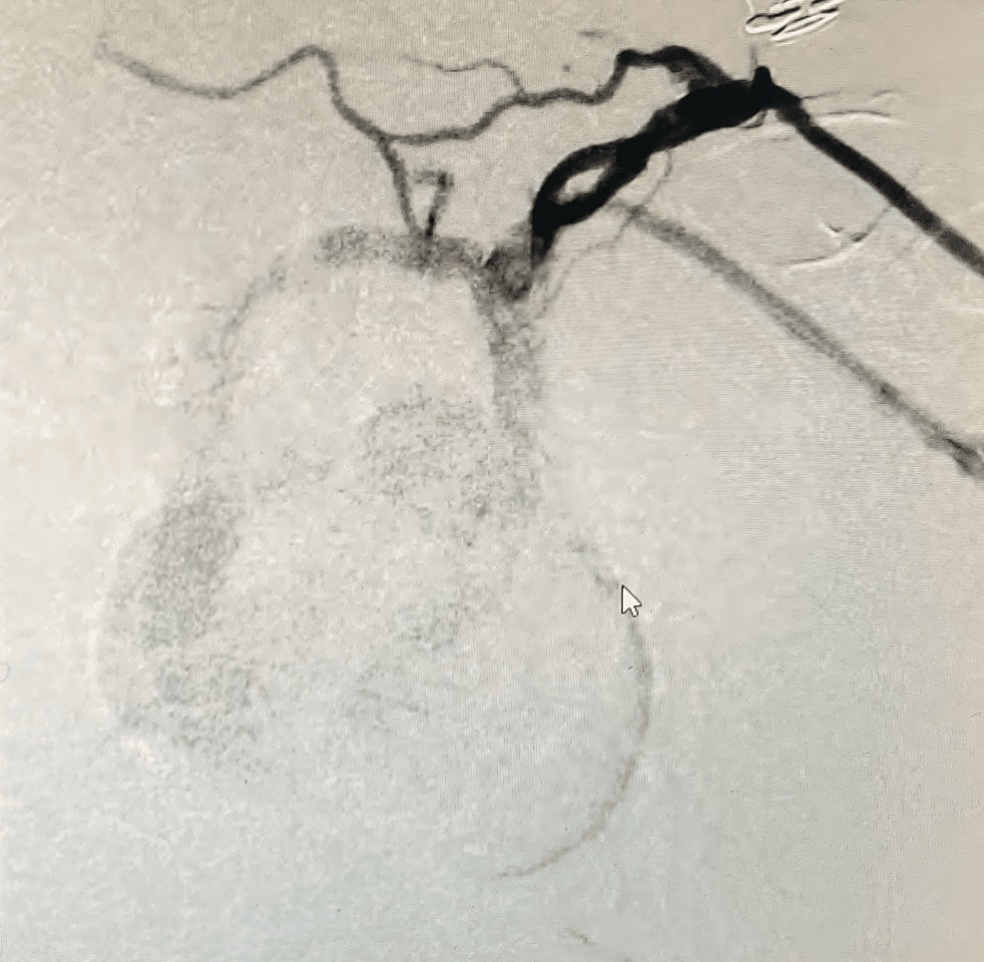
CT Angiography Showing Dilated UCS and PLSVC UCS: Unroofed coronary sinus; PLSVC: Persistent left superior vena cava The image has been taken by the authors.

CT angiography was done on the patient revealing dilated LA and left ventricle (LV), and a dual SVC was observed. The right SVC was observed to drain into the right atrium. The LSVC was seen to be extending caudally and opening into the CS, emptying into the LA (Figure 4). The appearance of a communicating vein was also seen connecting the two SVCs in the supra-aortic region (Figure 5).

**Figure 6:**
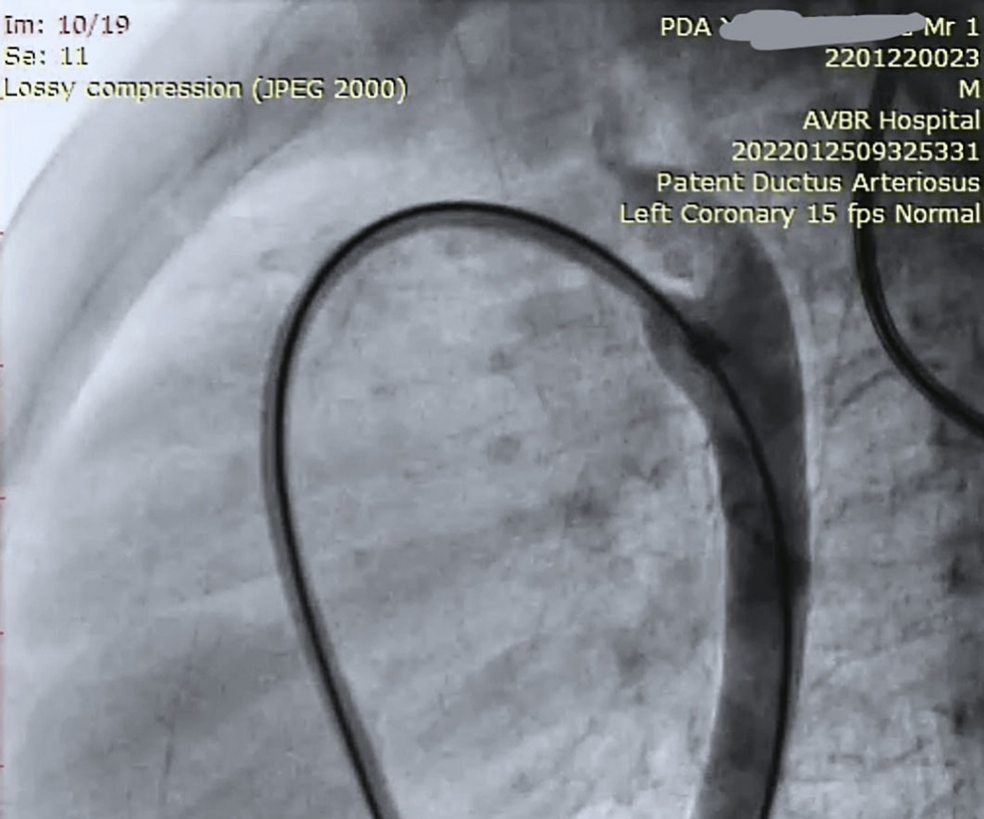
Standard Anterior-Posterior View Showing Duct Closure of the PDA PDA: Patent ductus arteriosus The image has been taken by the authors.

CT angiography was performed using device named cocoon for the closure of a large PDA as a surgical management in treating the patient (Figure 6).

## Discussion

The complete or partial absence of the roof of the CS is called an unroofed CS (UCS). This UCS connects the LA and the CS. The rarest type of atrial septal defect (ASD) is the UCS syndrome (UCSS) [9]. A frequent abnormal genetic cardiovascular condition in which a PLSVC pours across an enlarged CS into the right atrium. Association of an UCS with an LSVC terminating into the LA following interatrial communication is rare. Thus, the clinical consequences might be significant. The prevalence rate of PLSVC is 0.1% to 0.3% [7]. In our case, we have a PLSVC pouring into an UCS. A septal defect can be linked with a rise in incidence expectancy of systemic embolisation, pulmonary arterial hypertension, and brain abscesses [5]. 

The CS and the SVC develop from the common and anterior cardinal veins. At the end of the sixth month of the foetal life, the LSVC regresses. A minute remnant is a CS and the superior left intercostal vein. The left portion of the sinus venosus continues along due to the insufficiency of the left atriovenous fold, and LSVC terminates into the LA. In around 3-10% of people having genetic heart diseases, PLSVC is seen. LVCSs primarily terminate in the right atrium through the CS, but in around 10% of cases, they may terminate into the LA [10]. Association of LSVC and UCS in 75% of patients [11]. Atrio-ventricular septum defects, CS ostial atresia, and atrial appendage abnormalities are the various other cardiac anomalies related to UCS and PLSVC [12].

In the uterine life of the fetus, ductus arteriosus allows the flow of arterialized blood through the lungs from the placenta. After birth, the first breaths fill the lungs with air, and resistance of the pulmonary vascular structures decreases. Blood flows to the lungs from the right ventricle for its oxygenation causing the arterial oxygen tension to rise and the flow of blood decreases via the ductus arteriosus. This causes the construction of the duct. In healthy neonates who have completed the full term, the ductus arteriosus gets shut within 12-24 hours following birth. Within two-to-three weeks, permanent closure of the duct is complete. In infants born before the full term, in whom the ductus arteriosus fails to close promptly, pharmacologic or surgical closure modalities are used to cure side effects [13,14,15].

Several genetic abnormal conditions are linked with the development of PDA. They are trisomy of chromosomes 13, 18, and 21. Other conditions in which PDA can be seen are Holt-Oram syndrome, Charge syndrome, Noonan syndrome, DiGeorge, Thoracic Aortic Aneurysm (TAAD), and in hereditary PDA, and also in several types of genetic heart diseases [16,17]. Holt-Oram syndrome can be seen in the mutation of a gene on chromosome 12q2 [18]; Noonan syndrome is an autosomal dominant trait on chromosome 12q24 [19]; and Charge syndrome is a genetic anomaly caused due to the CHD7 gene mutation [20]. There are certain conditions which can present with UCS and these includes cardiac anomalies such as ASDs, atrial appendage abnormality, and CS ostial atresia {12].

The stenting of the ductus arteriosus is assisted by prostaglandins (PGEs) like PGE E2. After birth, the presence of patency in the ductus can lead to severe complications. They are congestive heart failure, pulmonary edema, necrotizing enterocolitis, pulmonary hemorrhage, intraventricular hemorrhage, kidney failure, and bronchopulmonary dysplasia [21]. Blood flows into the pulmonary circulation coming from the descending aorta through the ductus connecting left and right atria. This is known as left-to-right shunting. This shunting can result in pulmonary edema [22]. There is a rise in cardiac output as a compensatory mechanism due to this unwanted blood loss from the aorta during the diastole. In premature infants, the ability to surge stroke volume is limited, and as a result, the cardiac rate is high to rise the cardiac output. An increased risk for necrotising enterocolitis and renal failure is seen due to decreased flow of blood to the lower parts of the body [21,22].

CT angiography and imaging reconstruction can help with the magnificent anatomical description of the human heart, CS, and great vascular structures, supporting the specific prognosis of UCSS [23]. Deep structures like the pulmonary veins or the CS terminating into the posterior wall of the LA can be more accurately evaluated by cardiac MRI and transesophageal echocardiography [8].

## Conclusions

UCS is a rare genetic cardiac abnormality in which there is a communication between the CS and LA due to the presence of a LSVC along with an incomplete or complete loss of the CS roof draining into the LA. Its quite rare to have PDA along with UCS. Although the condition is rare, there could be a management of this syndrome. This condition had been surgically tackled in one sitting by re-routing of the LSVC to the right atrium using autologous pericardial patch along with ligation of PDA and ASD patch closure. If there is any connecting vein, as seen in this case, then LSVC can even be ligated. This case report will help readers gain knowledge about the rare findings of this particular syndrome and help medical professionals prognose, diagnose, and treat patients in an effective and faster manner.
